# Long‐Term Sequential Therapy for Pregnancy‐ and Lactation‐Associated Osteoporosis With Multiple Vertebral Fractures: A Case Report of Over 5 Years

**DOI:** 10.1155/crog/5696865

**Published:** 2026-06-12

**Authors:** Kaneyuki Tsuchimochi, Hiroe Tsuchimochi

**Affiliations:** ^1^ Department of Orthopaedic Surgery, Onga Nakama Medical Association Onga Hospital, Onga-cho, Onga-gun, Fukuoka, Japan; ^2^ Honjo Surgical and Orthopaedic Clinic, Kitakyushu, Japan

**Keywords:** denosumab, pregnancy- and lactation-associated osteoporosis, romosozumab, sequential therapy, teriparatide, vertebral fractures

## Abstract

**Background:**

Pregnancy‐ and lactation‐associated osteoporosis (PLO) is a rare condition that may cause multiple vertebral fractures during late pregnancy or the postpartum period. Evidence regarding treatment strategies remains limited, particularly regarding long‐term outcomes of sequential regimens combining anabolic and antiresorptive therapies.

**Case Presentation:**

A 37‐year‐old woman developed low back pain 3 months postpartum and was diagnosed with PLO accompanied by multiple acute and chronic vertebral fractures. Breastfeeding was discontinued, and initial treatment with vitamin D analogs and bisphosphonate therapy was selected based on clinical and patient‐specific considerations, including documented efficacy in PLO, the absence of reproductive safety concerns, and patient preferences. Despite early improvement, lumbar spine Z‐score remained persistently low at −3.0, and therapy was sequentially transitioned to daily teriparatide for 2 years, romosozumab for 1 year, and denosumab for maintenance. Over 5 years and 7 months, lumbar spine and femoral neck bone mineral density increased by 53.4% and 26.1%, respectively. No new fractures occurred, and serial bone turnover markers demonstrated appropriate anabolic and antiresorptive responses at each phase of therapy.

**Conclusion:**

This case highlights a successful long‐term sequential treatment strategy for PLO involving bisphosphonate, teriparatide, romosozumab, and denosumab in a patient without desire for further pregnancy, without reproductive safety constraints. The individualized sequential approach developed through patient education and shared decision‐making may help achieve sustained improvements in bone mineral density and prevent refracture in patients at very high fracture risk, particularly when an anabolic‐first strategy is not initially feasible.

## 1. Introduction

Pregnancy‐ and lactation‐associated osteoporosis (PLO) is a rare condition characterized by low‐trauma fragility fractures, most commonly affecting the vertebral bodies during late pregnancy or the postpartum period. The pathogenesis is multifactorial and incompletely understood, involving hormonal changes, disturbances in calcium metabolism, biomechanical stress, and genetic predisposition [1–3].

Although spontaneous bone mineral density (BMD) recovery may occur after weaning, patients with multiple vertebral fractures often experience persistent pain and remain at elevated fracture risk. Treatment is challenging due to the rarity of the condition, limited safety data in women of reproductive age, and the need for individualized management strategies [1–3].

Teriparatide, a recombinant parathyroid hormone analog, is one of the few anabolic agents approved for severe osteoporosis [4] and has also been reported in PLO [1–3, 5]. The importance of treatment sequence—initiating osteoanabolic therapy before antiresorptive agents—has been highlighted to maximize BMD gains in patients at high fracture risk [6].

Patients with multiple vertebral fractures are categorized as being at very high fracture risk, for whom rapid and maximal fracture risk reduction is recommended, often using an anabolic‐first approach according to the 2024 ASBMR/BHOF Task Force position statement [6]. However, actual treatment decisions may vary in clinical practice and often incorporate shared decision‐making based on patient preferences and reproductive plans.

In the present case, bisphosphonate therapy was initiated first based on clinical and patient‐specific considerations, followed by sequential therapy with teriparatide, romosozumab, and denosumab, achieving favorable outcomes over more than 5 years of follow‐up.

## 2. Case Presentation

**Figure 1:**
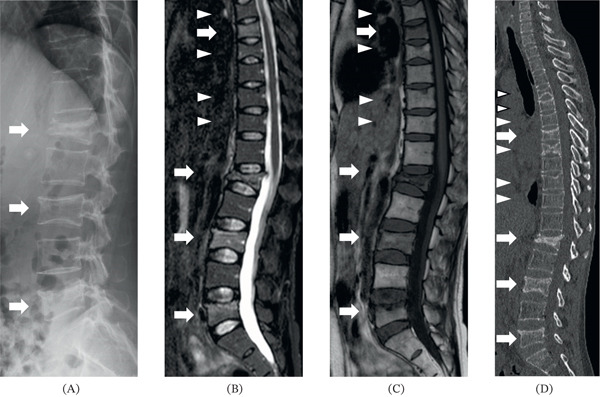
Imaging findings at the clinic visit and during emergency evaluation. (A) Lumbar radiographs obtained at the initial clinic visit showing fractures at L1, L3, and L5 (white arrows). (B) STIR sagittal MRI and (C) T1‐weighted sagittal MRI of the thoracolumbar spine performed after emergency transfer. Acute/subacute fractures with bone marrow edema are seen at T7, L1, L3, and L5 (white arrows) on the STIR sequence, whereas vertebral deformities at T6, T8, T10, and T11 show no marrow edema, consistent with chronic fractures (white arrowheads). (D) Whole‐spine CT (sagittal reconstruction) performed at the emergency hospital showing additional deformities at T4 and T5 (black‐outlined white arrowheads); their acuity could not be determined because these levels were outside the MRI field.

A 37‐year‐old woman developed low back pain while caring for her infant, 3 months after delivery, and presented to our clinic 10 days later (Day 0; all subsequent intervals are described relative to this visit). Lumbar radiography revealed vertebral fractures at L1, L3, and L5 (Figure 1A). Based on the clinical findings, a diagnosis of PLO was suspected, and breastfeeding was promptly discontinued. Treatment was initiated with a lumbar brace, loxoprofen sodium (60 mg three times daily), and alfacalcidol (1 *μ*g/day, oral).

Her symptoms worsened, necessitating transfer to the emergency department 12 days later (Day 12). Upon emergency transfer, the patient was unable to move due to severe pain (numeric rating scale [NRS] 8/10). She was treated with loxoprofen sodium (60 mg three times daily), fitted with a thoracolumbar orthosis, and maintained on bed rest for 2 weeks given her young age and severe pain. Pain improved to NRS 5/10 by 1 week after admission (Day 19), and further to NRS 1/10 by approximately 2 weeks (Day 27), at which point she was able to walk independently and was discharged home.

Magnetic resonance imaging (MRI) of the thoracolumbar spine showed acute or subacute fractures with bone marrow edema at T7, L1, L3, and L5. In contrast, vertebral deformities at T6, T8, T10, and T11 showed no bone marrow edema, consistent with chronic fractures. Notably, the patient had a history of a thoracic vertebral fracture identified incidentally on plain radiographs and MRI at another clinic 3 years earlier, performed for posterior neck and back pain that developed during stretching. The fracture was interpreted as chronic, and the exact timing of the original injury was unknown. No osteoporosis‐specific treatment or evaluation was initiated at that time. This prior fracture supports the interpretation of chronicity of some of the vertebral deformities observed on the current imaging (Figure 1B,C). Subsequent whole‐spine computed tomography (CT) revealed additional deformities at T4 and T5 (Figure 1D).

Her past medical history was notable for a low body mass index (BMI, 18.47 kg/m^2^), a history of smoking (5–10 cigarettes per day from age 20 until conception), and heavy alcohol consumption (14 or more drinks per week, until conception). She reported that her body weight had been low since adolescence. She had no history of eating disorders, endocrine or gastrointestinal disease, or menstrual irregularities. This was her first pregnancy, conceived spontaneously, delivered by cesarean section at 37 weeks due to breech presentation and a narrow pelvis following spontaneous rupture of membranes; birth weight was 2508 g. There was no family history of metabolic bone disease.

Laboratory evaluation was performed to exclude secondary causes of osteoporosis, including assessment of serum albumin, AST, ALT, *γ*GTP, creatinine, thyroid‐stimulating hormone, intact parathyroid hormone, estradiol, LH, FSH, calcium, phosphate, alkaline phosphatase, and 25‐hydroxyvitamin D. No specific secondary etiology was identified. Liver function tests showed no clinically significant abnormalities at baseline (Table 1) and remained normal throughout follow‐up, excluding alcoholic liver disease as a contributing factor. Alkaline phosphatase was mildly elevated, likely reflecting increased bone turnover in the context of multiple acute vertebral fractures, and estradiol was markedly reduced, consistent with the postpartum state. Bone turnover markers were markedly elevated, with high P1NP and TRACP‐5b indicating high bone turnover, likely driven in part by the acute bone repair response to multiple vertebral fractures. Undercarboxylated osteocalcin (ucOC) was elevated, consistent with vitamin K deficiency (Table 1). Menatetrenone (15 mg three times daily) was initiated on Day 15 in response to elevated ucOC and discontinued after 1 month. Normalization was confirmed 3 months after discontinuation without menatetrenone (ucOC 3.73 ng/mL).

**Table 1:** Baseline clinical and laboratory characteristics.

Parameter	Value	Unit	Reference range
Height	149.0	cm	
Weight	41.0	kg	
BMI	18.47	kg/m^2^	18.5–24.9 (normal)
Albumin	3.8	g/dL	4.1–5.1
AST	12	U/L	13–30
ALT	9	U/L	7–30
*γ*GTP	13	U/L	9–32
Cr	0.67	mg/dL	0.46–0.82 (female)
TSH	1.2	*μ*IU/mL	0.5–5.0
PTH‐intact	30	pg/mL	10–65
Estradiol (E2)	<10.0	pg/mL	
LH	2.1	mIU/mL	
FSH	5.9	mIU/mL	
Calcium	9.4	mg/dL	8.8–10.1
Phosphate	4.9	mg/dL	2.7–4.6
ALP (JSCC)	436	U/L	106–322 (JSCC method)
25‐OH Vitamin D	23.8	ng/mL	over 30 (sufficient); over 20 (insufficient per Japanese criteria)
Total P1NP	128.4	ng/mL	21.9–79.1 (female)
TRACP‐5b	1105	mU/dL	120–420 mU/dL
ucOC	5.14	ng/mL	< 4.5 (vitamin K deficiency indicator)

*Note:* Mildly reduced albumin, elevated bone turnover markers (P1NP and TRACP‐5b), vitamin K deficiency (ucOC), and markedly reduced E2 were observed. Phosphate was mildly elevated (4.9 mg/dL), and corrected calcium was within the normal range at presentation; no secondary etiology was identified. Albumin, calcium, and phosphate normalized by discharge (albumin 4.0 g/dL, calcium 9.5 mg/dL, phosphate 4.3 mg/dL).

Abbreviations: *γ*GTP, gamma‐glutamyl transpeptidase; ALP, alkaline phosphatase; ALT, alanine aminotransferase; AST, aspartate aminotransferase; BMI, body mass index; Cr, creatinine; E2, estradiol; FSH, follicle‐stimulating hormone; LH, luteinizing hormone; P1NP, procollagen type 1 N‐terminal propeptide; PTH, parathyroid hormone; TRACP‐5b, tartrate‐resistant acid phosphatase 5b; TSH, thyroid‐stimulating hormone; ucOC, undercarboxylated osteocalcin.

A diagnosis of PLO was made based on the clinical presentation, laboratory findings, and imaging results. As the patient consistently expressed no desire for further pregnancy throughout follow‐up, having been satisfied with one child, the possibility of future pregnancy was explicitly discussed given her age of 37 years, and she confirmed that she had completed childbearing. Pharmacologic therapy was therefore initiated without concerns related to future pregnancy. Oral alendronate (35 mg once weekly) was started on Day 10. Alfacalcidol was switched to eldecalcitol (0.75 *μ*g/day, oral) at Week 4 for long‐term supplementation. Calcium supplementation was not prescribed due to the risk of hypercalcemia associated with concurrent active vitamin D analog therapy. Standard dietary and lifestyle guidance was provided throughout the treatment course. Alendronate was switched to intravenous ibandronate (1 mg, once monthly) at Week 8.

Despite improvements in BMD with antiresorptive therapy, although femoral neck *Z*‐score improved, lumbar spine *Z*‐score remained persistently low at −3.0 (Table 2). Given its greater osteoanabolic potency and reported efficacy in PLO [5], daily teriparatide (20 *μ*g once daily, subcutaneous) was initiated at Week 68 and continued for 2 years, followed by romosozumab (210 mg once monthly, subcutaneous) for 1 year starting at Week 184. Thereafter, therapy was transitioned to denosumab (60 mg once every 6 months, subcutaneous) at Week 240 for ongoing maintenance, which has been continued for more than 1 year.

**Table 2:** Sequential changes in BMD and *Z*‐scores across treatment phases.

Phase	Wk	Treatment	*Δ*FN (%)	*Δ*LS (%)	C*Δ*FN (%)	C*Δ*LS (%)	FN *Z*‐score	LS *Z*‐score
1	25.0	BP	+5.3	+19.5	+5.3	+19.5	−2.4	−2.9
2	29.0	BP	+3.8	−2.3	+9.3	+16.8	−1.9	−3.0
3	47.7	BP → TPTD 33.7wk	+5.9	+9.9	+15.8	+28.3	−1.6	−2.4
4	63.0	TPTD	+8.2	+7.6	+25.3	+38.1	−1.5	−2.0
5	67.9	TPTD 7.3wk → Romo 48.3wk	−2.9	+8.4	+22.0	+46.5	−1.7	−1.3
6	60.3	Romo 3.7wk → Dmab	+3.7	+2.5	+26.1	+53.4	−1.5	−1.2

*Note:* Each phase corresponds to the interval between two consecutive BMD assessments. The column labeled “Wk (weeks)” indicates the duration between measurements. For each phase, the therapies administered, percentage changes (*Δ*) in femoral neck (FN) and lumbar spine (LS) BMD, cumulative percentage changes (C*Δ*), and corresponding Z‐scores are presented. Active vitamin D analogs were coadministered throughout bisphosphonate, romosozumab, and denosumab therapy, but were not administered during teriparatide therapy. Baseline *Z*‐scores prior to treatment initiation were −2.7 for FN and −3.8 for LS. *Z*‐scores shown in the table represent values measured at the end of each phase. *Z*‐scores of −2.0 or lower are considered below the expected range for age per ISCD criteria.

Abbreviations: BP, bisphosphonate; Dmab, denosumab; Romo, romosozumab; TPTD, teriparatide.

**Figure 2:**
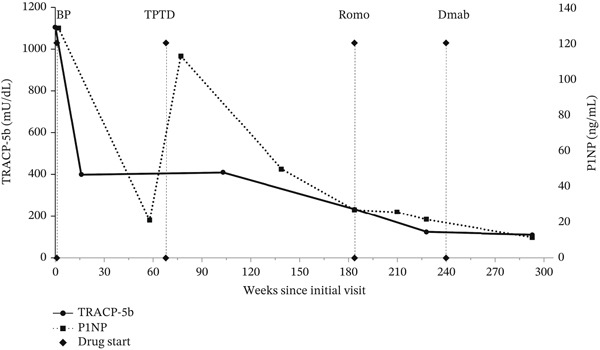
Serial changes in bone turnover markers. Serum P1NP (ng/mL) markedly increased during teriparatide therapy and was subsequently suppressed by antiresorptive treatment. TRACP‐5b (mU/dL) decreased during bisphosphonate therapy and showed further suppression during romosozumab and denosumab administration.

**Figure 3:**
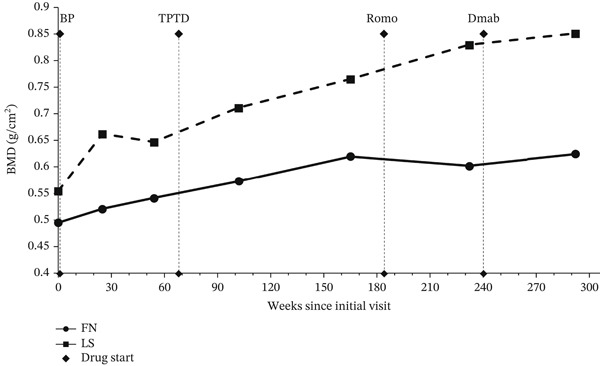
Serial changes in lumbar spine (LS) and femoral neck (FN) bone mineral density (BMD) and treatment timeline. Although LS‐BMD showed a marked initial increase followed by a slight decline during bisphosphonate therapy, it improved progressively with subsequent sequential treatment, with generally concurrent improvements in the femoral neck.

Serial bone turnover markers showed dynamic changes throughout the treatment course. P1NP increased markedly during the initial phase of teriparatide therapy, consistent with its anabolic action, whereas TRACP‐5b progressively declined (Figure 2). Sequential dual‐energy X‐ray absorptiometry (DXA) revealed marked gains in BMD, with lumbar spine and femoral neck values increasing by 53.4% and 26.1%, respectively, compared with baseline (Figure 3). Changes in BMD values and *Z*‐scores over the entire treatment period are summarized in Table 2.

Following discharge, the patient required assistance with childcare for approximately 3 months, after which she was able to resume full daily activities and independent childcare without pain. She has remained free of pain and functional limitations throughout the subsequent follow‐up period. No adverse effects attributable to early cessation of breastfeeding were observed, and the infant demonstrated normal growth throughout follow‐up. This case highlights the potential benefit of long‐term sequential pharmacologic therapy in patients with PLO at very high fracture risk, with substantial skeletal recovery demonstrated by both biochemical markers and densitometric outcomes.

## 3. Discussion

PLO is a rare condition, and optimal management remains uncertain because of limited evidence and the heterogeneous clinical presentations reported to date [1–3]. The rapid bone loss occurring in late pregnancy and during lactation is influenced by hypoestrogenism, elevated calcium demand, and increased bone resorption mediated by prolactin and parathyroid hormone‐related peptide (PTHrP) [1–3]. Although partial spontaneous recovery after weaning has been described, women presenting with multiple vertebral fractures often require pharmacologic intervention because of persistent pain and sustained fracture risk [1–3]. Recent reviews have highlighted the paucity of long‐term follow‐up data beyond 3 years as a critical evidence gap in PLO [2, 3].

In our patient, initial therapy with alfacalcidol followed by eldecalcitol and bisphosphonate resulted in substantial early BMD increases, likely reflecting the combined effects of lactation cessation, physiological rebound, vitamin D analogs, and antiresorptive therapy. Current guidelines recommend an “anabolic‐first” strategy for patients at very high fracture risk [6]; however, this evidence was not yet established at the time treatment was initiated. Bisphosphonate was selected as the initial therapy based on multiple considerations: its documented efficacy in PLO [7], the absence of reproductive safety concerns given the patient′s consistent wish to have no further pregnancies [2, 3], and her preferences regarding medication cost and route of administration. Bisphosphonates represent the most cost‐effective pharmacologic option for PLO, with substantially lower costs compared with teriparatide or romosozumab [8]. Reproductive counseling is an essential component of PLO management, particularly given the reproductive age of affected women. In this patient, breastfeeding was promptly discontinued at diagnosis to reduce PTHrP‐mediated maternal bone resorption [1, 3]. Contraception was confirmed throughout the treatment course, and the implications of bisphosphonate therapy—including long‐term skeletal retention and the risk of fetal exposure through placental transfer [8]—were discussed as part of informed consent. In women who have not completed childbearing, these considerations may favor an anabolic‐first approach over bisphosphonate initiation [2].

Transition to teriparatide was guided by goal‐directed treatment principles [6]: failure to achieve lumbar spine *Z*‐score normalization despite partial femoral neck recovery indicated that continued antiresorptive therapy was insufficient. Transition to romosozumab was additionally informed by emerging evidence demonstrating that this sequence substantially increases the probability of achieving BMD targets [9, 10]. Following successful teriparatide completion, romosozumab was used sequentially—rather than as a rescue agent for teriparatide intolerance or failure, as in previously reported PLO cases [11, 12]—capitalizing on evidence that this sequence achieves greater BMD gains than the reverse order [13]. This intensive strategy—comprising two sequential osteoanabolic agents, long‐term denosumab maintenance, and planned bisphosphonate therapy following denosumab discontinuation to mitigate rebound bone loss [14]—was warranted given her baseline Z‐scores of −2.7 and −3.8, which fell substantially below the expected range for age per ISCD criteria and within the more severe range reported in PLO [2, 3, 15].

The total lumbar spine BMD increase (53.4% over 5 years and 7 months) compares favorably with previously reported PLO cases with long‐term follow‐up, including a 21.4% increase over 5 years with vitamin D analog therapy [16] and a 44.8% increase over 12 years with bisphosphonate treatment [7]. Long‐term follow‐up data for sequential therapy combining teriparatide, romosozumab, and denosumab in PLO remain limited, and these data may contribute to the long‐term evidence base identified as lacking in recent reviews [2, 3]. The absence of new fractures during extended follow‐up further supports the effectiveness of the sequential approach, and is favorable compared with the subsequent fracture rate of 24.3% reported over a median of 6 years in a large PLO cohort [17].

The pathogenesis of PLO is multifactorial, encompassing hormonal changes during pregnancy and lactation, increased calcium demand, and patient‐specific risk factors. This patient had a low BMI (18.47 kg/m^2^) with a self‐reported history of low body weight since adolescence, a history of smoking, and heavy alcohol consumption (14 or more drinks per week, until conception), all of which were confirmed patient‐specific risk factors. Chronically low body weight since adolescence is considered the primary contributor to impaired peak bone mass accrual and low baseline BMD in this patient, and the cumulative impact of these confirmed risk factors may have contributed substantially to her elevated fracture risk. An underlying genetic susceptibility cannot be excluded, as variants in LRP5, WNT1, and COL1A1/2 have been reported in up to half of women with PLO [15]. It should be noted that the present case involves a patient who had completed childbearing and did not desire further pregnancy, and therefore does not reflect the full complexity of PLO management in women of reproductive intent. Nonetheless, the long‐term follow‐up data provided here address an evidence gap identified in recent reviews [2, 3], and the sequential treatment approach described may offer insights applicable to a broader range of patients with PLO at very high fracture risk.

In summary, individualized management guided by goal‐directed treatment principles and regular monitoring of BMD and bone turnover markers are important for achieving sustained improvements in BMD and fracture prevention in PLO at very high fracture risk.

## 4. Conclusion

This case demonstrates successful long‐term management of PLO with multiple vertebral fractures using a sequential regimen of bisphosphonate, teriparatide, romosozumab, and denosumab over 5 years and 7 months. An individualized, sequential approach developed through patient education and shared decision‐making may help achieve sustained improvements in BMD and prevent refracture in patients at very high fracture risk, particularly when an anabolic‐first strategy is not initially feasible.

## Author Contributions

Kaneyuki Tsuchimochi: conceptualization, diagnosis, treatment planning, primary clinical management and long‐term patient follow‐up (hospital and clinic), data collection and analysis, interpretation of imaging and laboratory data, and manuscript preparation. Hiroe Tsuchimochi: supplementary clinical follow‐up at the clinic, patient care support, and manuscript review.

## Funding

No funding was received for this manuscript.

## Disclosure

All authors reviewed and approved the final manuscript.

## Ethics Statement

Ethical approval was not required for this case report according to the policies of Onga Nakama Medical Association, Onga Hospital, which does not mandate review for individual case reports or case series.

## Consent

Written informed consent was obtained from the patient for publication of this case report and the accompanying images.

## Conflicts of Interest

The authors declare no conflicts of interest.

## Data Availability

The data that support the findings of this case report are available from the corresponding author upon reasonable request, but are not publicly available due to privacy restrictions.
